# Myelomastocytic transformation in chronic myeloid leukemia blast phase: A case report

**DOI:** 10.1007/s12308-025-00653-7

**Published:** 2025-08-02

**Authors:** Abdulrahman F. Al-Mashdali, Feryal Ibrahim, Samah Kohla, Ibrahim Ganwo, Susanna Akiki, Mohammed Abdulgayoom, Mohamed A. Yassin

**Affiliations:** 1 Department of Hematology and Bone Marrow Transplant, National Center for Cancer Care and Research (NCCCR), Hamad Medical Corporation, Doha, Qatar; 2 Department of Laboratory Medicine and Pathology, Hamad Medical Corporation, Doha, Qatar

**Keywords:** Chronic myeloid leukemia, Myelomastocytic leukemia, Blast phase, Acute myeloid leukemia

## Abstract

Myelomastocytic leukemia (MML) presenting as a blast phase manifestation of Chronic Myeloid Leukemia (CML) is exceptionally rare, with limited documented cases in the literature. Understanding its distinct clinicopathologic features and treatment outcomes is crucial for optimal patient management. A 45-year-old male with a history of CML since 2016, previously treated with imatinib and dasatinib, presented after treatment interruption with leukocytosis (WBC 27.3 × 103/μL) and 58% circulating blasts showing metachromatic granulation. Bone marrow examination revealed 30% blast cells with strong CD117 and tryptase positivity. Flow cytometry identified two distinct populations: 7% myeloblasts and 27% immature myeloid cells with bright CD117 expression. BCR-ABL1 rearrangement was confirmed with a ratio of 112% (IS). The patient received combination therapy with standard “3 + 7” induction chemotherapy and dasatinib. Despite complications of febrile neutropenia, the post-induction bone marrow examination demonstrated achievement of complete morphologic remission. This case highlights the successful initial treatment of myelomastocytic transformation in CML blast phase using intensive combination therapy. The detailed morphologic, immunophenotypic and molecular characterization provides valuable insights into this rare entity, while the favorable initial response supports an aggressive treatment approach. Long-term follow-up and further studies are needed to establish optimal treatment strategies.

## Introduction

Chronic myeloid leukemia (CML) is a distinct myeloproliferative neoplasm characterized by the BCR-ABL1 fusion gene, resulting from acquired balanced reciprocal genetic translocation, t(9;22) (q34;q11.2) happening in haemopoietic stem cells that result in the so-called Philadelphia (Ph) chromosome. The disease typically progresses through three clinical phases: chronic, accelerated, and blast phase with the blast phase being defined by greater than 20% blasts in peripheral blood or bone marrow. While myeloid (70%) and lymphoid (20–30%) blast transformations are most common, rare variants can occur [1–3]. Myelomastocytic leukemia (MML) represents a rare entity traditionally associated with advanced myeloid neoplasms, particularly refractory anemia with excess blasts and AML. It is characterized by elevated numbers of immature atypical mast cells (> 10% metachromatic blasts) without meeting the full criteria for systemic mastocytosis, notably lacking CD2/CD25 expression, D816V KIT mutation, and focal dense mast cell infiltrates [4]. The occurrence of myelomastocytic blast transformation in CML blast phase (CML-BP) is exceptionally rare, with only four documented cases in the medical literature prior to our report. This case represents the fourth reported instance of this unique phenotype, making it a significant contribution to the existing knowledge base [5]. This unusual presentation poses significant diagnostic and therapeutic challenges.

## Case presentation

A 45-year-old male, initially diagnosed with CML-chronic phase in 2016, presented to our institution in January 2025. His medical history revealed initial treatment with imatinib, which was later switched to dasatinib due to resistance. He achieved MMR four months after starting dasatinib and maintained the response for a further year. In February 2019, the patient left Qatar for his home country, where financial constraints led to treatment discontinuation. He returned to Qatar in January 2025, initially seeking medical attention at a private center for a one-week history of fever and upper respiratory tract infection. He had no symptoms or signs of mast cell activation, including skin reactions like itching and flushing or gastrointestinal issues like diarrhea and vomiting. Initial laboratory investigations revealed leukocytosis with WBC 30 × 103/μL, prompting referral to our institution for further evaluation and management.

**Fig. 1:**
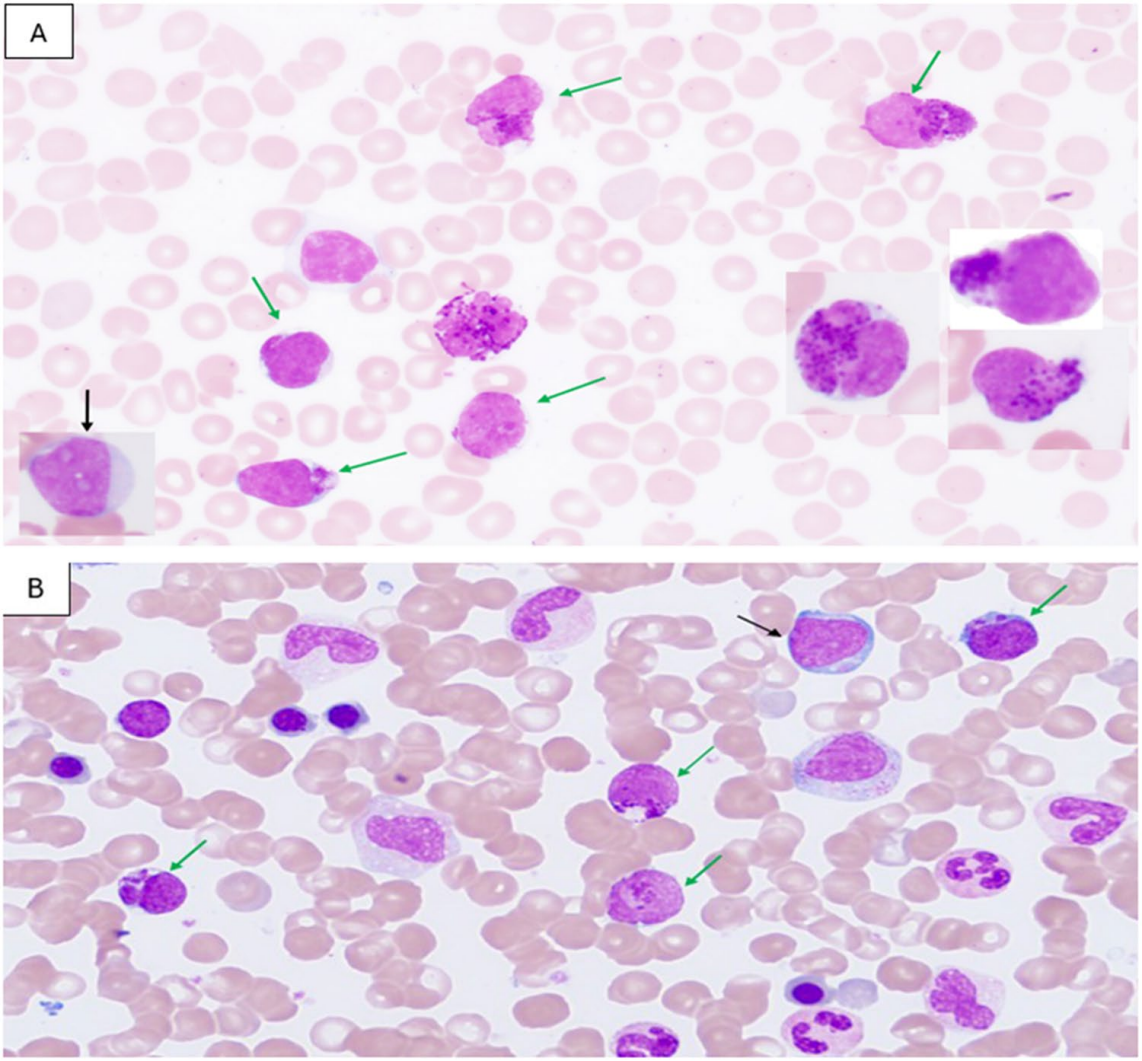
**A** Peripheral blood shows many circulating immature cells with fine chromatin, some with metachromatic coarse granules (green arrowed myelomastocytic blasts) with right insert shows another three blasts, and myeloblast without granules (black arrowed, lower left insert). **B** Bone marrow aspirate shows similar blasts with metochromatic granules (myelomastocytic blasts, green arrowed) and blast without granules (myeloblast, black arrowed). × 1000. Wright’s Stain

A comprehensive evaluation at our institution on January 19, 2025, revealed significant hematological abnormalities. The complete blood count showed WBC 27.3 × 103/μL, hemoglobin 12.6 g/dL, platelets 239 × 103/μL, and ANC 1.5 × 103/μL. Abdominal ultrasound revealed no hepatosplenomegaly. Peripheral blood examination was notable for leukocytosis with approximately 58% circulating blasts, where the majority (50%) demonstrated coarse metachromatic to basophilic granulation, while a smaller proportion (8%) appeared agranular (Fig. 1A).

Bone marrow examination revealed hypercellular smears infiltrated with approximately 30% blast cells, morphologically similar to those observed in peripheral blood, including 8% agranular blasts and 22% granulated blasts (Fig. 1B), set against a background of granulocytic cells showing dysplastic features and adequate but dysplastic erythropoiesis.

**Fig. 2:**
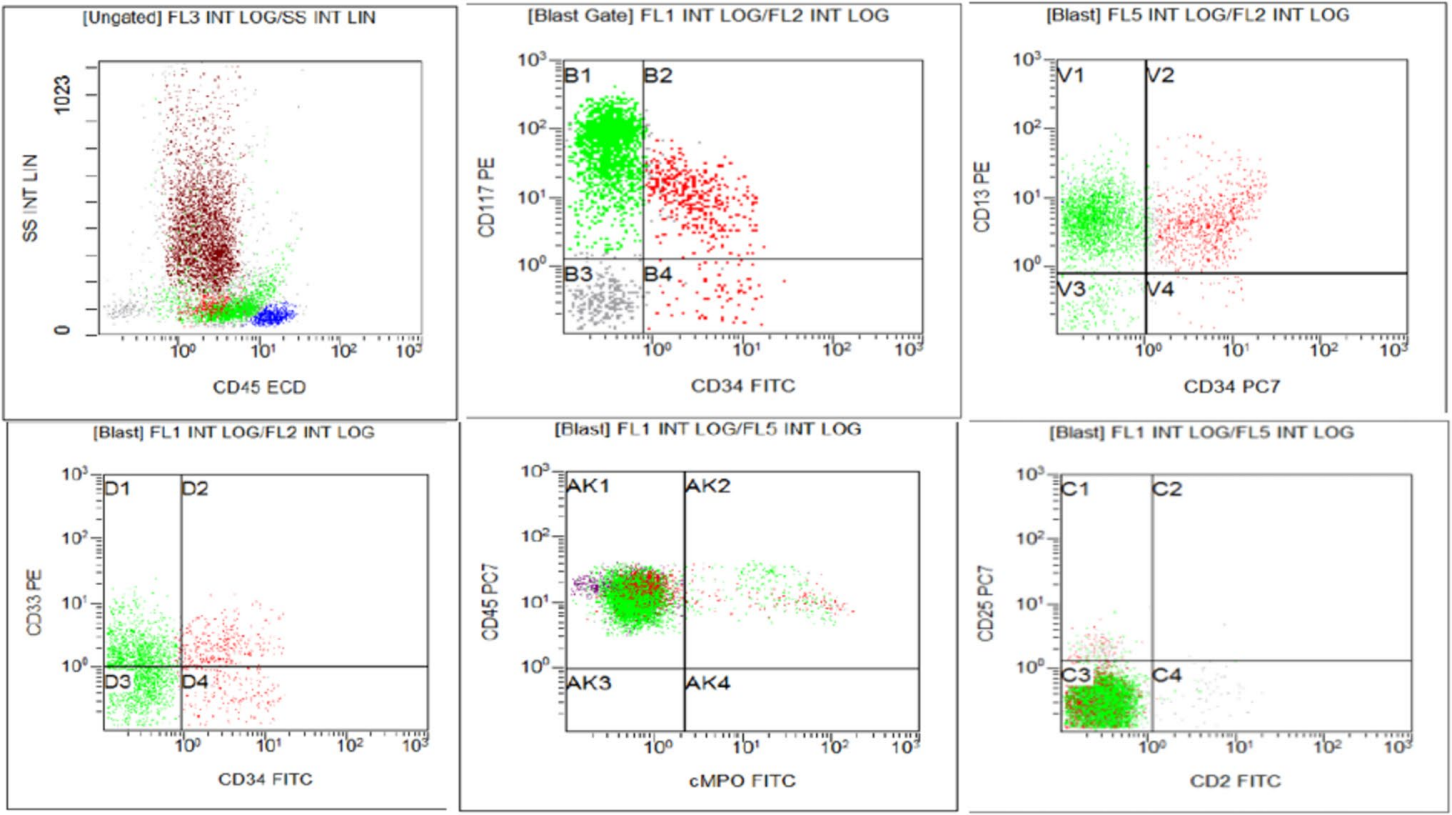
Flow cytometry immunophenotyping showed 2 populations of CD45 dim blasts. One population (green colored) was strongly positive for CD117 (myelomastocytic blasts), with a small population (colored red) was weakly positive for CD117 (myeloblasts). Myelomastocytic blasts were negative for CD34, while myoblasts were positive. Both blasts population were positive for CD13, partial CD33 and negative for cMPO, CD25 and CD2

At this stage, morphology suggested a differential diagnosis of either a basophilic blast transformation or aggressive systemic mastocytosis such as mast cell leukemia. Meanwhile, flow cytometry analysis was performed on the bone marrow aspirate and identified 7% myeloblasts expressing CD34, CD117, HLA-DR, CD13, and CD123, with partial expression of CD33 and CD36, and aberrant expression of CD7 and CD4. Additionally, 27% of the immature cells were detected expressing bright CD117, CD13, and CD123, with partial CD33, CD11c, and aberrantly expressing CD4 and partial CD7. Both populations were negative for CD2 and CD25 (Fig. 2). The bright expression of CD117 excludes the possibility of basophilic blasts, as basophilic blasts are essentially CD117-negative. Serum tryptase level was within the normal limit (10 ng/mL).

**Fig. 3:**
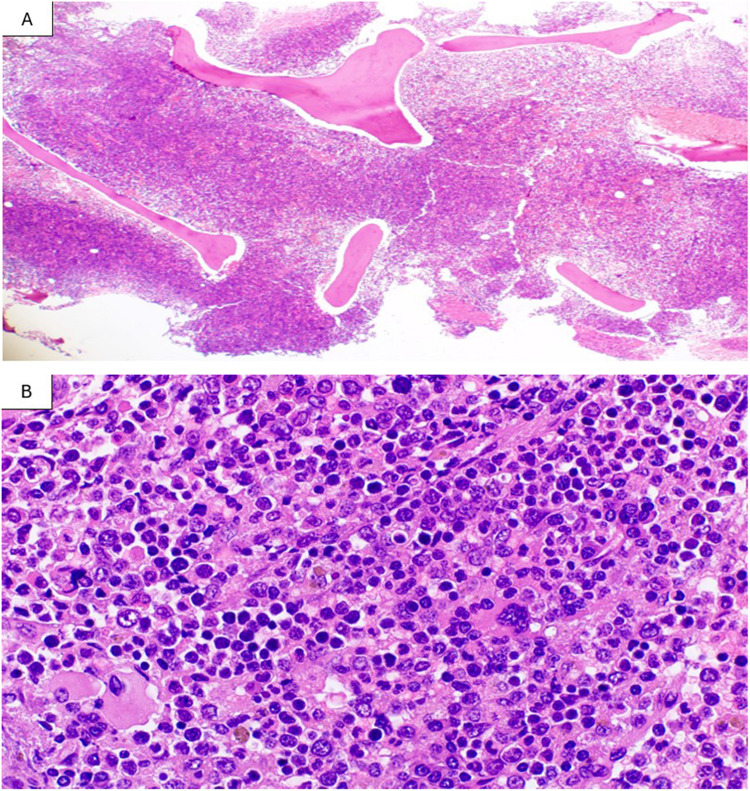
**A** Bone marrow biopsy shows remarkably hypercellular marrow (100% cellularity) × 40. H&E. **B** Bone marrow biopsy shows adequate megakaryocytes, hyperactive erythropoiesis and granulopoiesis and interstitial increase primitive looking cells. × 600. H&E

**Fig. 4:**
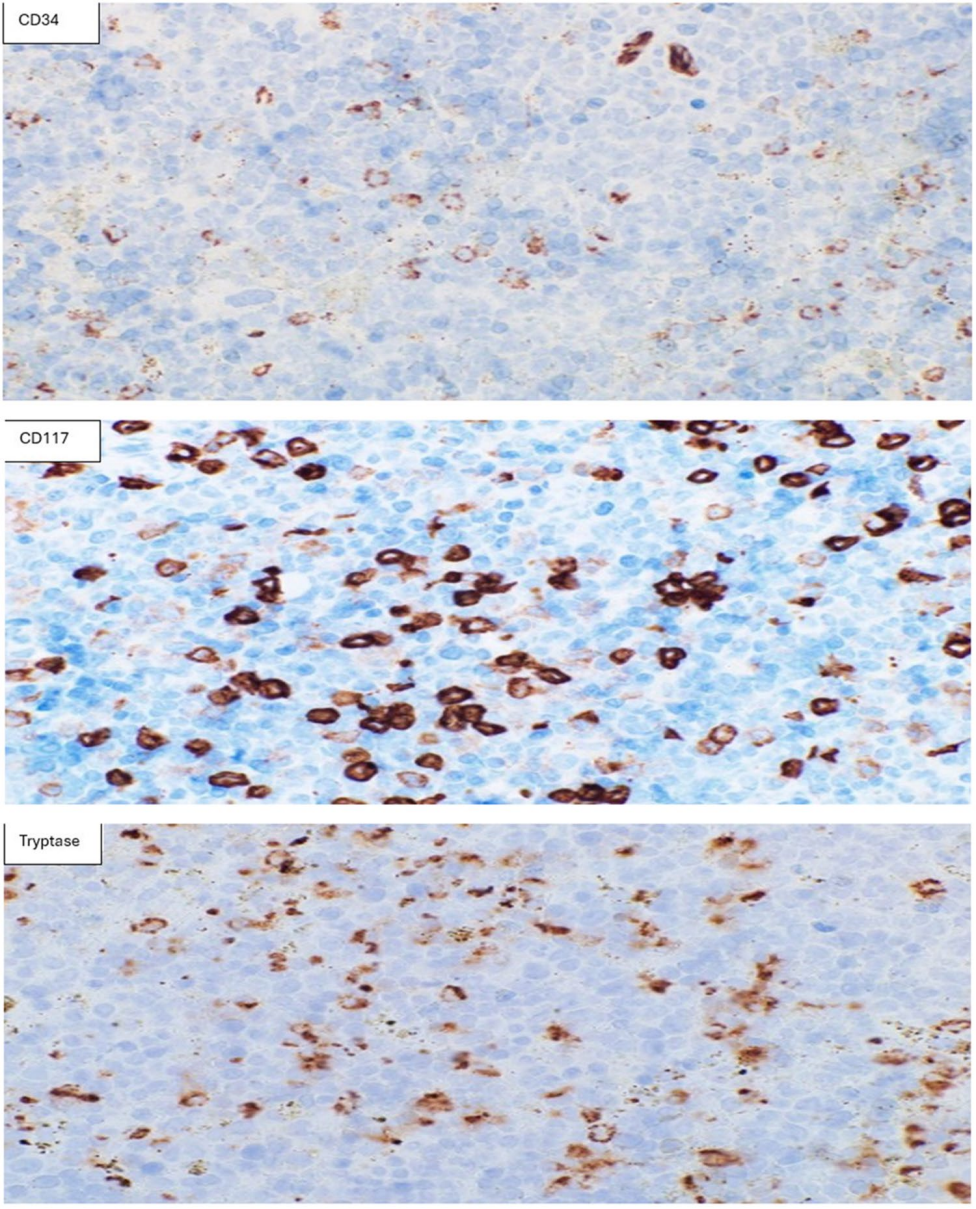
Immunohistochemical stains; CD117 shows an increase in CD117 + blasts (some were strongly positive (myelomastocytic blasts) and others weakly positive (myeloblasts). Tryptase stain, many blasts were positive in scattered pattern without focal dense aggregates. CD34 was positive in the small population of the blasts (was roughly estimated at 8–10%)

The bone marrow biopsy demonstrated remarkable hypercellularity (almost 100%) with hyperactive erythropoiesis and granulopoiesis, intermingled with increased primitive-looking cells that were dispersed throughout the marrow (Fig. 3A, B). CD117 immunohistochemistry clearly highlighted many strongly positive blasts, in addition to some weakly positive blast cells. Because of morphology and the strong CD117 positivity, tryptase immunostaining was performed and revealed increased tryptase-positive atypical mast cells in a scattered pattern without focal dense aggregates. CD34 highlighted increased CD34-positive blasts, roughly estimated at 8–10% (Fig. 4), and the negative expression of CD25 was also confirmed by IHC.

Fluorescence in situ hybridization (FISH) confirmed the presence of *BCR::ABL1* rearrangement in 80% of analyzed cells, and karyotype revealed 46,XY,t(9;22)(q34;q11.2)/46,XY [4]. Quantitative RT-qPCR demonstrated *BCR::ABL1* positivity with a *BCR::ABL1* to *ABL1* percentage ratio of 112% (IS). Next-generation sequencing (NGS) showed a clinically significant *RUNX1* frameshift mutation (*p.R346Pfs*) at 38% VAF, an *ASXL1* nonsense variant (VUS) at 40% VAF, and *BCR::ABL1* (*e13a2*) fusion (18.4% reads), while mutation *D816V* in *KIT* was not detected.

Compiling these findings, a diagnosis of chronic myeloid leukemia in myeloid blast phase with morphology and immunophenotype of myelomastocytic leukemia was made. Following a multidisciplinary team review, the patient was started on standard “3 + 7” induction chemotherapy (idarubicin × 3 days + cytarabine × 7 days) combined with dasatinib. The patient completed two cycles of therapy, complicated by febrile neutropenia requiring supportive care, including broad-spectrum antimicrobials. Post-induction evaluation confirmed complete remission (CR) on bone marrow assessment, along with a major molecular response (MMR; BCR::ABL1 ≤ 0.1% IS) on RT-qPCR, indicating an excellent treatment response. Given this favorable outcome, the patient is now planned for allogeneic stem cell transplantation (allo-SCT) from a matched sibling donor with curative intent.

## Discussion

Myelomastocytic leukemia (MML) is a very rare type of myeloid leukemia characterize by a prominent differentiation towards mast cell lineage with increase in metachromatic blasts and immature mast cells (promastocytes), in the context of an advanced myeloid neoplasm, usually MDS with excess of blasts, AM L, or an accelerated/blast phase of MPN or MDS/MPN. MML is characterized by increased immature myelomastocytic cells (> 10% in bone marrow or peripheral blood) expressing CD117 and tryptase with metachromatic granules. This rare condition typically presents with a specific immunophenotypic profile (CD117 +  +, tryptase +  +, CD11b −, CD123 −, CD2 −, CD25).While MML features can occur in various myeloid neoplasms, its presentation as a blast phase manifestation of CML is exceptionally rare [6, 7].

The diagnostic distinction between MML and related mast cell disorders requires careful consideration of specific pathologic features. While MML is characterized by diffusely distributed immature cells lacking CD2 and CD25 expression and the c-KIT D816V mutation, other entities in the differential diagnosis present with distinct characteristics: systemic mastocytosis and SM-AHN typically show dense clusters of mature mast cells, mast cell leukemia demonstrates a predominance of immature mast cells, and all these classic mast cell disorders share common features including CD117 and tryptase expression, aberrant CD2 and/or CD25 expression, and the presence of c-KIT D816V mutation in most cases [4, 7, 8]. Understanding these distinguishing features is crucial for accurate diagnosis and appropriate therapeutic selection, particularly in challenging cases such as myelomastocytic transformation in CML blast phase.

Treatment strategies for myelomastocytic leukemia remain largely empirical due to its rarity, with limited data available to guide clinical decision-making. Historical outcomes with conventional cytoreductive therapy and supportive care alone have been disappointing, with patients experiencing notably short survival durations. However, more aggressive therapeutic approaches have shown promising results in a small number of documented cases: one patient achieved complete remission with only TKI, while two additional patients attained complete remission through a combined approach of chemotherapy followed by allogeneic stem cell transplantation [9]. These outcomes, though limited in number, suggest that more intensive treatment strategies, particularly those incorporating allogeneic stem cell transplantation, may offer better outcomes than conventional approaches, though the optimal treatment algorithm remains to be defined through larger studies and longer follow-up periods. The role of novel targeted therapies and immunotherapeutic approaches in this rare entity also warrants further investigation.

Table 1 summarizes the four previously reported cases in the literature resembling our patient. Our case represents a striking example of myelomastocytic transformation in the blast phase of chronic myeloid leukemia (CML), characterized by key diagnostic findings: elevated immature myelomastocytic cells (50% in peripheral blood, 22% in bone marrow) with metachromatic granules, along with strong CD117 positivity and tryptase expression.

**Table 1:** Summary of the reported cases in the literature

Author (year)	Age/sex	Initial treatment	Time to BP	BM Myeloid blast %	IBM MCS %	Cytogenetic findings	KIT D816V mutation	Therapy given	Outcome
Vigil et al. (2011) [10]	50/M	Not mentioned	Initial extramedullary CML-BP (orbital mass & bone lesions)	Not mentioned	Sheets of aberrant CD25 + cells	Cryptic BCR::ABL1	Negative	Dasatinib + low-dose Ara-C	CR (morphologic/cytogenetic) for 11 months
Martinez et al. (2019) [11]	29/M	Imatinib → dasatinib → nilotinib	7 years	29%	46% aberrant CD25 +	Failed karyotyping	Not reported	Chemotherapy (3 + 7) + bosutinib	Early death (CNS bleeding)
Song et al. (2020) [5]	74/M	Imatinib → nilotinib	1 year	5%	38% aberrant CD25 +	Complex t(9;22)	Negative (T315I, ASXL1 & RUNX1 mutations)	Ponatinib	Achieved CR

This case differs from classic mast cell disorders due to the diffuse infiltration of immature cells (rather than dense mast cell aggregates), the presence of *BCR::ABL1* rearrangement, and a unique immunophenotypic profile revealing two distinct populations: conventional myeloblasts (7%) and immature myeloid cells with bright CD117 expression (27%).

Treatment with induction chemotherapy combined with tyrosine kinase inhibitor (TKI) therapy (dasatinib) achieved complete morphologic remission, reinforcing emerging evidence supporting intensive therapeutic strategies. This report provides valuable insights into the need for vigilant monitoring of atypical transformations in CML, particularly after treatment interruption. It also raises critical questions regarding optimal post-remission therapy, the potential role of allogeneic stem cell transplantation, and the necessity of long-term follow-up to assess response durability.

## Conclusion

This case represents a rare instance of myelomastocytic transformation in CML blast phase, successfully treated with combined intensive chemotherapy and TKI therapy. The achievement of complete morphologic remission supports an aggressive treatment approach in such cases. By detailing the clinicopathological features and demonstrating a successful initial treatment response, this case significantly enriches the limited literature on this rare entity. It offers practical guidance for managing similar cases while underscoring key areas for further research.

## Data Availability

No datasets were generated or analysed during the current study.
